# Insights into physical activity promotion among Australian chiropractors: a cross-sectional survey

**DOI:** 10.1186/s12998-024-00543-2

**Published:** 2024-06-14

**Authors:** Matthew Fernandez, Katie de Luca, Craig Moore, Simon D. French, Paulo Ferreira, Michael Swain

**Affiliations:** 1 Discipline of Chiropractic, Faculty, School of Health, Medical and Applied Sciences, Central Queensland University, Brisbane, Australia; 2 Department of Chiropractic, Faculty of Medicine, Health and Human Sciences, Macquarie University, Sydney, Australia; 3 Sydney Musculoskeletal Health, School of Health Sciences, Charles Perkins Centre, Faculty of Medicine and Health, University of Sydney, Sydney, NSW Australia

**Keywords:** Chiropractic, Physical activity, Promotion, Health promotion

## Abstract

**Background:**

Despite the well-known benefits of physical activity, physical inactivity is presently a global health pandemic. Allied healthcare providers, such as chiropractors, knowingly recognise the importance of physical activity and are prepared to routinely discuss and/or counsel patients on this topic; however, little is known about Australian chiropractors in the physical activity setting. Our aim was to explore and identify factors associated with physical activity promotion among Australian chiropractors, including their knowledge of the physical activity and sedentary behaviour guidelines and their own levels of physical activity.

**Methods:**

From February to May 2021, a convenience sample of Australian chiropractors completed an online survey. Items assessed by Likert scale included: physical activity promotion frequency, with the type, quantity, barriers, perceptions, and feasibility. We asked questions about their familiarity with, and knowledge of, Australian Physical Activity and Sedentary Behaviour Guidelines, chiropractors’ own physical activity, and whether the chiropractors met activity guidelines. Survey responses were descriptively reported. Univariable logistic regression models explored factors explaining frequent physical activity promotion.

**Results:**

Of 217 respondents, 64% reported that they frequently (≥ 70%) recommended a more physically active lifestyle. Only 15% often performed pre-exercise screening, 73% frequently prescribed resistance exercise, 19% reported time as the most frequent barrier, while 37% reported being not at all familiar with the guidelines. Univariable logistic regression models found male chiropractors were more likely to promote physical activity, [odds ratio (OR) = 2.33; 95% confidence interval (CI): 1.32–4.12)], while chiropractors who frequently treat children 0–3 years (OR = 0.5; 95% CI: 0.28–0.87), children 4–18 years (OR = 0.42; 95% CI: 0.21–0.86), and pregnant women (OR = 0.5; 95% CI: 0.26–0.94) were less likely. Chiropractors were more likely to promote physical activity if they were familiar with the activity guidelines (OR = 2.9; 95% CI: 1.32–6.41), were confident promoting (OR = 11.6; 95% CI: 1.37–98.71) and prescribing physical activity programs (OR = 4.5; 95% CI: 2.03–9.99).

**Conclusion:**

Most chiropractors confidently and regularly integrate physical activity into practice. Yet, despite acknowledging its importance, one third of chiropractors reported poor knowledge of the Physical Activity and Sedentary Behaviour Guidelines. Identifying barriers to the awareness, and implementation of physical activity guidelines should be further explored within chiropractic clinical settings.

**Supplementary Information:**

The online version contains supplementary material available at 10.1186/s12998-024-00543-2.

## Background

Being physically active is important for health and wellbeing across the lifespan. In their most recent physical activity (PA) guideline recommendations, the World Health Organization (WHO) explicitly recommend that children, adolescents, adults, older adults, pregnant and postpartum women and those with chronic conditions or disability increase their PA [1]. Despite these recommendations, almost 1.5 billion people globally are considered physically inactive [2]. In Australia specifically, 55% of adults (aged 18–64) do not meet PA/sedentary behaviour (SB) guidelines, while only 12% of children and 2% of adolescents met their PA/SB recommendations [3]. The WHO specifically recommends that individuals undertake 150–300 min of moderate intensity PA (i.e., brisk walking) or 75–150 min of vigorous intensity PA (i.e., running) weekly [1]. For health benefits, children and adolescents should undertake 60 min daily of moderate-to-vigorous intensity PA [1]. Additionally, all age groups should embark on a resistance training program twice weekly [1].

Given the complexity of physical inactivity, a ‘whole of systems approach’ is now advocated [4]. Namely, various sectors that influence individual PA approaches are now targeted. Briefly, the systems approach aims to scale-up policy actions by creating active societies, active environments, active people, and active systems. Collectively, these approaches target communities, transport, urban planning, health care, sport, and workplaces [4].

Within this ‘whole of systems approach’ lies the healthcare sector, where allied health professionals (AHPs) play an important role in the promotion of population PA [4]. As a trusted health source, professionals such as nurses [5] and general practitioners [6], as well as AHPs like exercise physiologists, physiotherapists [7] and podiatrists [8] all reportedly promote PA to their patients, acknowledging it as part of their role. However, while a high proportion of AHPs report being confident in providing specific advice and that they regularly counsel and suggest PA programs, few are familiar with or have specific knowledge of the PA/SB guidelines [9].

Like other AHPs, chiropractors are well positioned to promote PA. In Australia, the chiropractic profession represents a substantial component of the allied healthcare system. Approximately 16% of the population consult chiropractors within a twelve month period [10], estimated at more than 20 million patient consultations per year [11]. Chiropractors are trained in the diagnosis and management of musculoskeletal conditions, which may increase the risk of developing chronic diseases [12]. Hence, many people seeking chiropractic care report comorbid chronic diseases such as symptoms of depression or anxiety [13]. The promotion of a physically active lifestyle by chiropractors may benefit the course of musculoskeletal conditions and reduces the risk of multiple chronic or longer-term diseases [14].

A recent systematic review found chiropractors recognise the importance of PA promotion and are prepared to routinely discuss and/or counsel patients on this health topic [15]. Additionally, previous research has shown that 85% of Australian chiropractors report often discussing PA as part of their patient management [16]. While these findings provide data and insights into practice, the nature of these PA discussions with patients remains unclear. To date there has been no in-depth analysis of how Australian chiropractors incorporate and promote PA, nor their general knowledge of the Australian PA/SB guidelines [3]. The aim of this study was to explore factors associated with the promotion of PA by chiropractors, including the frequency, type, quantity, barriers, perceptions, and feasibility of promotion, as well as their knowledge of PA/SB guidelines.

## Methods

This study was approved by Macquarie University Human Research Ethics Committee (Project ID 9044): 52,020,904,424,064.

Through convenience sampling, our cross-sectional online survey was conducted between February and May, 2021. Registered Australian chiropractors were recruited through an advertisement distributed via email to members of the two Australian chiropractic associations (Australian Chiropractors’ Association [*n* = 3,723] and Chiropractic Australia [*n* = 1,125]), members of the Sports Chiropractic Australia special interest group (*n* = 286) and members of two Australian Alumni associations (Macquarie University and Central Queensland University) using a link to an online questionnaire. Additionally, the study was promoted through social media outlets (e.g., Facebook) to attract non-association member participation. Prior to participation, potential participants were asked to confirm their practice and whether they were currently registered within Australia in any setting, e.g., private practice, community health, academia etc. Potential participants received recruitment materials including eligibility criteria, what is involved and were provided a link to the survey. Potential participants were informed that 10 min was required to complete the survey. To facilitate online survey response rates, participants had the opportunity to win one of five $100 sports vouchers.

The online questionnaire (Supplement A) was based on a previously validated questionnaire used in allied health [7] and general practice populations [6]. Minor amendments were made to the instrument to contextualise the questions to the chiropractic setting. The online survey included questions related to chiropractors’ demographic (age, gender) and practice characteristics (years in practice, hours worked per week, number of patient visits, characteristics of patients and practice). Using a five-point Likert scale for most survey items (i.e., 1 = Strongly disagree to 5 = Strongly agree), chiropractors were asked about the frequency of PA promotion to their patients, as well as the type, quantity, barriers, perceptions, and feasibility of PA promotion. Chiropractors’ familiarity and knowledge of Australia’s PA/SB Guidelines for Australian Adults [17] were explored, with multiple response descriptions provided, and chiropractors selected the response that best reflected their understanding of current PA/SB recommendations for adults aged 18–64 years. Chiropractors were asked questions relating to their own individual PA engagement, i.e., compare your current level of PA to other Australians of your sex and similar age. Chiropractors were also asked whether they accumulated 150 to 300 min of moderate intensity PA or 75 to 150 min of vigorous intensity PA, or an equivalent combination of both moderate and vigorous activities. Chiropractors were also asked if they had engaged in muscle strengthening activities at least twice per week in the last 6-months. We explored the frequency of PA promotion among chiropractors using the question “What percentage of your patients did you recommend having a more physically active lifestyle (apart from therapeutic /rehabilitative exercise) in the last month?”.

Sample size estimates were based on the total number of registered chiropractors in Australia in September 2019 (*n* = 5,556). Using an online sample size calculator, we chose a 95% confidence level, 0.5 standard deviation, and a margin of error of 5%. The sample size formula comprises:

$$\eqalign{{\rm{n = (z - score}}{{\rm{)}}^{\rm{2}}} & \times \;{\rm{standarddeviation}} \cr & \; \times \;{\rm{(1 - standard}}\;{\rm{deviation)}}\;{\rm{/}}\;{{\rm{(margin}}\;{\rm{of}}\;{\rm{error)}}^{\rm{2}}} \cr}$$

$${\rm{n = (1}}{\rm{.96}}{{\rm{)}}^{\rm{2}}} \times \;{\rm{0}}{\rm{.5}}\; \times \;{\rm{(1 - 0}}{\rm{.5)/(0}}{\rm{.05}}{{\rm{)}}^{\rm{2}}}$$

The sample size calculated was 385 participants.

The survey was critically evaluated and independently pre-tested by three sessional chiropractic academics with more than 10 years clinical experience: i.e., two practising chiropractors who had completed a Master of Research, and the third was a practising chiropractor and PhD candidate. A further three registered chiropractors also independently pre-tested the questionnaire to assess usability, understandability, and consistency. After one round of pre-testing, recommendations were integrated into to the original survey, specifically wording, and ordering of questions.

The Research Electronic Data Capture (REDCap) system was utilised to distribute the online surveys and for data collection [18]. Data were inspected, with responses checked for duplicate IP addresses prior to finalising data for analysis. Data were exported to IBM SPSS (Version 26.0) where missing data were checked and given that no question in the survey was compulsory, unanswered questions from incomplete surveys were excluded. Data were cleaned, with descriptive statistics generated for all variables. Chiropractors’ responses to questions about practice characteristics, frequency of PA promotion, and their knowledge of the Australian PA/SB guidelines for adults were reported as counts and proportions. Likert response options were dichotomised into ‘frequently-every time’=0 and ‘never-sometimes = 1’. Likewise, factors that might explain frequent PA promotion by chiropractors were dichotomised into responses to reflect opinions ‘neutral-dissenting’=0 and ‘affirmative’=1. This was conducted for variables related to type, quantity, barriers, perceptions, and feasibility of PA promotion, and familiarity of Australia’s PA/SB guidelines for Australian adults. Univariable logistic regression models were used to identify candidate factors that may explain frequent PA promotion among chiropractors. The estimate of all logistic regression analyses was reported as odds ratios (OR) with 95% confidence intervals (CI) that were calculated using Wilsons Score Interval for proportions and p-values. All quantitative analyses were conducted using IBM SPSS Statistics for Windows, Version 26.0. Armonk, NY: IBM Corp.

## Results

The sample comprised 217 chiropractors (59.3% Male, 40.7% Female). Table 1 shows chiropractors’ number of years in practice, number of hours worked per week and number of patient visits per week, as well as other relevant practice characteristics. Table 1 further describes the personal characteristics of all chiropractors registered in Australia at the time of the survey being distributed. Due to the recruitment methods, an accurate response rate was not possible to determine, but is estimated to be 5%, based on the total number of chiropractors affiliated with professional bodies in Australia.

**Table 1:** Practitioner, patient, and practice characteristics of Australian chiropractors

Practitioner	Fernandez 2023 (*n* = 217)	* Adams 2017 (*n* = 2,005)	- CBA Jan-Mar 2021 (*n* = 5,948)
Male	59.3	62.4	58.6
**Age**			
< 34 years	37.8	-	34.5
35–44 years	25.8	-	26.3
45–54 years	20.3	-	21.6
55–64 years	12.9	-	12.0
> 64 years	2.3	-	5.6
**State**			
NSW/ACT	52.9	-	33.7
NT	0.5	-	0.4
QLD	17.3	-	15.7
SA	6.3	-	6.4
TAS	1.4	-	1.1
VIC	13.9	-	26.5
WA	6.3	-	12.7
**Private practice**			
Hours worked per week	28.4	27.3	-
No of patients treated per week	71.4	87.3	-
**Patient subgroup**			
Older people (65 years and over)	82.5	73.5	-
Children (4 to 18 years)	75.1	53.2	-
Children (up to 3 years)	42.9	30.1	-
Athletes or sports people	82	49.5	-
Post-surgical rehabilitation	35	6.4	-
Pregnant women	69.1	36.7	-

- Age (years), n (%)

- Data for total population of registered Chiropractors in Australia: Chiropractic Board of Australia (CBA) Registrant Data, 2021 [32]

* Data from Adams et al., Australian chiropractic workforce study considered representative of the population [11]

In total, 64.1% (95% CI: 57.5-70.2%) of chiropractors reported that, in the past month, they recommended ≥ 70% of their patients to have a more physically active lifestyle (Table 2). Regarding chiropractors’ practice, 14.7% (95% CI: 10.5-19.9%) performed pre-exercise screening of patients prior to recommending PA (Supplementary Table 1). Resistance exercise was the most often prescribed PA by chiropractors (72.9%; 95% CI: 66.7-78.5%). Similarly, 75.3% (95% CI: 69.3-80.7%) of chiropractors reported ‘frequency’ as the most often prescribed exercise component of the ‘FITT’ exercise formula: Frequency, Intensity, Time and Type. A lack of time was the most frequent barrier for chiropractors to promote PA (18.8%; 95% CI: 13.9-24.5%). Approximately 5% of chiropractors reported a lack of counselling skills and a lack of remuneration as barriers for promoting PA, while 11% thought PA promotion would not change patient behaviour (Supplementary Table 1).

**Table 2:** Frequency of Australian chiropractors recommending a more physically active lifestyle

	*n*	% (95% CI)
Never	0	0%
Rarely, in less than 10% of patients in the last month	7	3.2% (1.5-6.2%)
Occasionally, in about 30% of patients in the last month	31	14.3% (10.1-19.4%)
Sometimes, in about 50% of patients in the last month	40	18.4% (13.7-24%)
Frequently, in about 70% of patients in the last month	67	30.9% (25-37.2%)
Usually, in about 90% of patients in the last month	44	20.3% (15.3-26%)
Every time	28	12.9% (8.9-17.8%)

Regarding perceptions of PA, 96.6% (95% CI: 93.5%, 98.5%) of chiropractors felt confident in providing general advice to patients about a physically active lifestyle, and 97.6% (95% CI: 94.9-99.1%) considered discussing the benefits of a physically active lifestyle as an important part of their clinical role. Most chiropractors (94.3%; 95% CI: 90.5-96.8%) agreed that breaking-up sedentary behaviour as often as possible, with any form of movement of any intensity was important for good health. Whereas 63% (95% CI: 56.3-69.3%) agreed that short bursts of exercise that gets your body warm, sweaty, and breathing heavily was good for health. Conversely, more than half of chiropractors (60.5%; 95% CI: 53.8-66.9%) were neutral or disagreed with the statement that good health only requiring 30 min of brisk walking on most days (Supplementary Table 2).

Established community-based PA programs, e.g. walking groups, was considered the most highly feasible facilitator by chiropractors (69.8%; 95% CI: 63.2-75.7%). Brief exercise counselling integrated into consultations, as well as the distribution of PA education resources, was also considered highly feasible for PA promotion by > 50% of chiropractors (Supplementary Table 3). Approximately one third (36.6%; 95% CI: 30.2-43.3%) of chiropractors reported being ‘not at all familiar’ with the Australian PA/SB guidelines for adults, with less than 10% being ‘extremely familiar’ with current recommendations (Supplementary Table 4).

Almost half of the chiropractors (46.3%; 95% CI: 39.5-53.2%), were able to accurately identify all key components associated with Australia’s current PA/SB Guidelines for adults aged 18–64 years. In total, 20.7% (95% CI: 15.6-26.7%) of chiropractors were not sure of the correct PA/SB recommendation when presented with different options (Supplementary Table 5). Almost three quarters of chiropractors (74.8%; 95% CI: 68.4-80.4%) believed they were more active when compared to other Australians of the same sex and similar age. Similarly, > 80% of chiropractors reported meeting the aerobic component of the guideline recommendations and > 75% reported meet the resistance training activities, as per the PA/SB guidelines in the last 6 months (Supplementary Table 6).

Table 3 outlines the results of pairwise logistic regression models constructed to evaluate chiropractor characteristics associated with encouraging patients to be more physically active, referenced against those who infrequently recommend PA. Male chiropractors were more likely to promote PA than females (OR = 2.33; 95% CI: 1.32–4.12), while chiropractors who frequently treated children, aged 0–3 years (OR = 0.5; 95% CI: 0.28–0.87), or children aged 4–18 years (OR = 0.42; 95% CI: 0.21–0.86), and pregnant women (OR = 0.5; 95% CI: 0.26–0.94) were less likely to promote PA.

**Table 3:** Exploratory logistic regression models identifying Australian chiropractor characteristics associated with encouraging patients to be more physically active, referenced against those who infrequently recommend physical activity

			OR	95% CI	*P*-Value
**Practitioner and Patients**					
Gender (Male)			2.33	1.32–4.12	**0.003**
Children (up to 3 years)			0.5	0.28–0.87	**0.015**
Children (4–18 years)			0.42	0.21–0.86	**0.017**
Pregnant women			0.5	0.26–0.94	**0.032**
**Practice**					
Private practice			0.32	0.13–0.76	**0.01**
Both private practice and academia			2.67	1.11–6.43	**0.028**
**How frequently do you recommend or prescribe the following**					
How often do you perform pre-exercise screening (e.g., baseline bodyweight, heart rate and blood pressure etc.) on your patients prior to recommending physical activity?			3.52	1.3–9.56	**0.014**
Aerobic exercise (i.e., endurance training). *n* = 213			5.11	2.72–9.6	**< 0.0001**
Resistance exercise (i.e., strength training). *n* = 214			4.59	2.42–8.7	**< 0.0001**
**How frequently do you determine or quantify your prescription of physical activity under the FITT principles (Frequency, Intensity, Time and Type)**					
Frequency (how often). *n* = 215			2.33	1.24–4.38	**0.009**
Intensity (how hard). *n* = 214			2.06	1.17–3.65	**0.013**
Time or duration (how long). *n* = 211			2.17	1.22–3.88	**0.009**
Type (aerobic/cardio, strength, endurance). *n* = 213			3.08	1.69–5.62	**< 0.0001**
**To what extent do you agree or disagree with the following statements**:					
Good health requires adding large muscle group strengthening activities (such as resistance or weight training) a few times per week. *n* = 209			2.47	1.21–5.05	**0.013**
As a chiropractor, I feel confident in giving general advice to patients about a physically active lifestyle. *n* = 207			11.65	1.37–98.71	**0.024**
As a chiropractor, I feel confident in suggesting specific physical activity programs to my patients. *n* = 210			4.5	2.03–9.99	**< 0.0001**
**What kind of physical activity promotion is or would be feasible for you to deliver to your patients (beyond prescribing therapeutic /rehabilitative exercise)?**					
a. Brief exercise counselling integrated into your regular consultations. *n* = 208			4.55	1.92–10.75	**0.001**
b. Separate one-on-one consultations. *N* = 208			2.24	1.25–4.01	**0.007**
c. Group sessions. *n* = 208			1.84	1.02–3.31	**0.043**
d. Distribution of educational resources (e.g., Brochures). *n* = 205			2.88	1.28–6.48	**0.011**

Statistically significant findings highlighted in bold (p value < 0.05)

Chiropractors who were familiar with the Australian PA/SB guidelines (OR = 2.9; 95% CI: 1.32–6.41), and confident in providing general advice to patients about a physically active lifestyle (OR = 11.6; 95% CI: 1.37–98.71) and specific PA programs to patients (OR = 4.5; 95% CI: 2.03–9.99), were more likely to promote PA. Of these, brief exercise counselling integrated into regular consultations was considered most feasible (OR = 4.55; 95% CI: 1.92–10.75), while chiropractors who encouraged PA with their patients were two-to-three times more likely to advocate all elements of the “FITT” exercise formula. Non-significant findings evaluating chiropractor characteristics associated with encouraging patients to be more physically active, referenced against those who infrequently recommend PA, can be found in Supplementary Table 7.

## Discussion

Approximately two-thirds of our convenience sample of chiropractors frequently recommended that patients have a more physically active lifestyle. While some key findings suggest chiropractors are confident and promote PA, our findings suggest that areas for improvement remain in this aspect of clinical practice. These areas not only pertain to certain identified barriers that need to be overcome to promote PA, but also that many chiropractors report being unfamiliar with PA/SB guidelines, especially in youthful and pregnant populations.

Our finding that > 60% of chiropractors recommended PA in the last month is consistent with studies conducted among Australian general practitioners [6], physiotherapists [5], podiatrists [8] and other AHPs [7, 9]. However, only a smaller percentage of chiropractors (15%) frequently perform pre-exercise screening prior to making PA recommendations, compared to a higher percentage of physiotherapists (34%) and general practitioners (41%) [19]. This may relate to a potential lack of referral pathways for chiropractors if high risk patients are identified. It may also be due to a lack of training or knowledge, or due to the additional time required to apply risk stratification screening [20]. Considering exercise is safe for most people [20], a fast and simple form of pre-screening may be achieved by implementing particular PA assessment tools. For instance, the post-2015 American College of Sports Medicine questionnaire now primarily screens the individual’s current PA level [20]. As chiropractors knowingly obtain PA information from patients [15], implementing tools like the validated General Practice Physical Activity Questionnaire (GPPAQ) can enhance the screening process [21]. The GPPAQ can briefly assess PA levels in routine practice, grouping patients into inactive-to-active categories that can be easily conveyed to patients [21]. This can facilitate the PA conversation for time-pressured chiropractors in practice.

Although aerobic activity is forefront in many PA guideline recommendations, almost three quarters of chiropractors in our study reported resistance exercise as their most often prescribed PA. While the reasons for favouring muscle-strengthening activities are not clear, it may relate to enablers of resistance training prescription such as the convenience of using only one’s body weight as resistance. Alternatively, it may also be that chiropractors place less emphasis of on aerobic training prescription due to traditional barriers, such as limited access to or resistance to joining a gym facility and risk of injury with fitness training equipment. It may be that chiropractors are comfortable prescribing resistance exercise for specific musculoskeletal conditions and recognise the broader benefits of resistance training, including positive gains in cognitive function, cancer survival and metabolic health as well as reduced mortality risk [22]. Resistance training performed either in isolation or concurrently with aerobic training is considered at least equal, or even superior, for health gains when compared to aerobic training alone [22]. Chiropractors favouring the promotion of resistance training is in contrast to Australian physiotherapists, who reportedly require further education on resistance training activities in relation to PA/SB guideline implementation [5].

While a high proportion of Australian chiropractors in our sample were confident in providing PA advice and acknowledged it is part of their clinical role, only half accurately identified key components associated with PA/SB guidelines. It is reasonable to speculate that despite chiropractor’s limited knowledge of the guidelines, confidence providing PA may stem from their own personal PA engagement, which is known to positively influence clinical PA attitudes and practices [23]. In our study > 75% of chiropractors believed they engaged in sufficient resistance training and > 80% identified achieving sufficient aerobic training participation for their age and sex. Nevertheless, almost 40% of chiropractors were ‘not at all familiar’ with the PA guidelines. Almost half of those surveyed were not convinced good health is achieved with 30 min of moderate intensity exercise daily, despite the moderate PA recommendations by the WHO [1]. Despite this lack of guideline knowledge, our findings, overall, are consistent with AHPs, both in Australia [5, 8], and internationally, who are aware of PA guideline existence, but have relatively poor knowledge surrounding the recommended key components.

Despite some gaps in understanding the PA guideline, almost 90% of chiropractors surveyed agreed that ‘any or all activity’ counts. Consistent with the WHO 2020 PA recommendations [1], this ties in with health benefits that knowingly accumulate in low doses of very brief PA bouts [24]. Recent evidence suggests the effects of 3-to-4 bouts of intense, incidental activity, e.g. running to catch a bus or train, or stair climbing, may be comparable to the health benefits attained by achieving 75–150 min of PA per week [24]. Health benefits could therefore ensue among chiropractic patients who view current PA guidelines as unachievable or those who go from being inactive to making relatively small PA changes over time. For chiropractors, promoting this form of PA is advantageous, given that it is incidental, feasible, time efficient, and unlikely to be planned.

Chiropractors who often recommend PA to their patients are half as likely to recommend PA to pregnant women. This finding is significant, given the health benefits of PA or exercise during pregnancy is considerable, including less weight gain, reduced risk of gestational diabetes, preeclampsia, and improved mood [25]. The lack of PA promotion to pregnant women is underscored by > 50% of chiropractors in our study being unfamiliar with the PA/SB guidelines. It is therefore plausible that pregnant women could be receiving little-to-no advice and possible misinformation pertaining to PA by chiropractors. Evidence indicates that only 30% of pregnant women in Australia were considered sufficiently active [26]. PA advice is likely to be well-received by pregnant women from AHPs, thus training chiropractors in brief PA counselling and providing further education on the current guideline recommendations for pregnancy is essential. As a minimum, chiropractors can provide quality PA information and help correct myths, misconceptions and confusion about PA during and after pregnancy, as well as encouraging exercise as a safe activity for pregnant women.

Similarly, chiropractors who often recommend PA to their patients are 50% less likely to recommend PA to children and adolescents. In Australia, paediatric patients comprise a significant portion of many chiropractors’ practices, with 9% of all visits being from children [27]. The reason for the poor promotion of PA of these patients is unclear and may relate to chiropractors limiting their scope of care mainly to musculoskeletal complaints like spinal pain, but specific non-musculoskeletal conditions, like infantile colic in babies for this patient population [28]. PA/SB guidelines in Australia specifically target the needs of babies, young children and adolescents, given the positive benefits to growth and development, as well as social, cognitive, and psychological welfare benefits [29]. Yet, children and adolescents in Australia are not meeting PA/SB recommendations, which has been further compounded by the COVID-19 pandemic, dominated by school closures, home schooling and a loss of school-based PA opportunities [30]. Considering this, the importance of minimising sedentary behaviour is underscored, and chiropractors are well placed to facilitate this in younger patients. Collectively, this may include specific PA recommendations during recreational and leisure activities (i.e., play), promoting active transport strategies (i.e., walking and cycling) for adolescents and more reading and storytelling activities with reduced screen time in children [31]. Notably, PA recommendations should be safe, enjoyable and matching children and adolescent abilities [29].

To the authors’ knowledge, this is the first cross-sectional survey that presents a thorough exploration of chiropractic practice with respect to PA promotion in Australia. Accordingly, we are less uncertain with chiropractors’ PA/SB guideline knowledge and barriers, as well as their perceptions and feasibility to promote PA. This provides a platform for further research enquiry and practice implementation.

Our study is subject to several limitations that need acknowledgement and cautious interpretation, especially the generalisability of our findings. We included a limited, non-random sample size of 217 clinicians, which limits generalisability to the Australian chiropractic profession. Only personal characteristics (i.e., age categories) were representative of the broader population. Larger samples are therefore necessary for the findings from our sample to be considered applicable to the wider population of Australian chiropractors. Our survey was only offered online, making it difficult to track how clinicians chose to participate or not, hence it is not possible to calculate the response rate. However, 5,948 chiropractors were registered in Australia (based from the Chiropractic Board of Australia’s quarterly data report in January to March 2021) [32], and our recruitment strategy targeted professional bodies reaching approximately 60% of the profession, thus our estimated response rate of 217 respondents is estimated at 5%. Online surveys likely exclude those lacking electronic devices to respond. There is also the potential for recall bias due to the use of self-report measures, as well as social desirability bias, response bias (i.e., sports voucher incentive) and selection bias (i.e., chiropractors interested in PA more likely to complete the survey). This possibly over or under-estimated the proportions and ORs reported in our study. In this light our work can be considered exploratory, and while p-values provide important insights into statistical significance of observed associations, our findings should be considered in the broader context, i.e., examining the reported CIs for additional interpretation. Importantly, our survey was distributed to chiropractors during the COVID-19 pandemic which may have affected their PA thoughts and promotional capacity at the time. Our cross-sectional design does not allow causal inferences and we cannot discount chiropractors’ attitudes and behaviours changing over time, particular as new PA knowledge comes to light and is implemented into practice.

PA as a ‘vital sign’ is an indicator of general physical condition. Thus, the impact of implementing the use of assessment tools by chiropractors to screen patients and starting the PA conversation is important and should be further explored. Further training to enhance PA knowledge, skills and implementation are essential, so chiropractors can effectively use PA/SB guidelines, particularly for pregnancy and youthful populations. Of note, chiropractors were familiar with, and recommended, community-based programs such as walking groups, but to what extent these were recommended along with referral patterns is unknown. Also, the experiences of the Australian chiropractors and the pubic with chiropractors and their PA promotional activities should be explored.

## Conclusion

In our study, chiropractor respondents were confident promoting PA despite one third of chiropractors reporting poor knowledge of the Australian PA/SB guidelines. Most believed PA promotion is an important part of their clinical role. To best fulfill and maximise implementing PA promotion into practice, further investigation is needed to address the barriers identified to improving PA advice, as well as enhancing and implementing PA/SB guideline knowledge, particularly PA promotion in pregnancy and youth populations.

### Electronic supplementary material

Below is the link to the electronic supplementary material.

Supplementary Material 1

Supplementary Material 2

Supplementary Material 3

Supplementary Material 4

Supplementary Material 5

Supplementary Material 6

Supplementary Material 7

Supplementary Material 8

## Data Availability

All data generated or analysed during this study are included in this published article.
