# Supplementary material for: Insights into physical activity promotion among Australian chiropractors: a cross-sectional survey
Source: Chiropr Man Therap. 2024 Jun 14;32:22. doi: 10.1186/s12998-024-00543-2 (PMC11179190; doi:10.1186/s12998-024-00543-2)
Supplement: Supplementary file 1 — Supplementary Material 1 [file 12998_2024_543_MOESM1_ESM.docx]

**Supplementary Table 1. Frequency of physical activity promotion activities and barriers for Australian chiropractors.**

|  | **Never** | **Rarely** | **Sometimes** | **Often** | **Very Often** |
| --- | --- | --- | --- | --- | --- |
| **How often do you perform pre-exercise screening (e.g., baseline bodyweight, heart rate and blood pressure etc.) on your patients prior to recommending physical activity? n=217** | | | | | |
|  | 28.6% (22.9%-34.8%) | 38.2% (32%-44.8%) | 18.4% (13.7%-24%) | 8.8% (5.5%-13.1%) | 6% (3.4%-9.7%) |
| **How frequently do you recommend or prescribe the following** | | | | | |
| Aerobic exercise (i.e., endurance training). n=213 | 1.9% (0.6%-4.4%) | 15.5% (11.1%-20.8%) | 34.7% (28.6%-41.3%) | 34.3% (28.1%-40.8%) | 13.6% (9.5%-18.7%) |
| Resistance exercise (i.e., strength training). n=214 | 0.5% (0.1%-2.2%) | 5.1% (2.8%-8.7%) | 21.5% (16.4%-27.4%) | 48.1% (41.5%-54.8%) | 24.8% (19.3%-30.9%) |
| Flexibility training (i.e., stretching). n=212 | 0.5% (0.1%-2.2%) | 8% (4.9%-12.2%) | 21.2% (16.1%-27.1%) | 36.3% (30.1%-42.9%) | 34% (27.8%-40.5%) |
| Balance training. n=214 | 4.7% (2.4%-8.1%) | 11.7% (7.9%-16.5%) | 36% (29.8%-42.6%) | 33.6% (27.6%-40.2%) | 14% (9.9%-19.1%) |
| **How frequently do you determine or quantify your prescription of physical activity under the FITT principles (Frequency, Intensity, Time and Type)** | | | | | |
| Frequency (how often). n=215 | 5.1% (2.7%-8.7%) | 4.7% (2.4%-8.1%) | 14.9% (10.6%-20.1%) | 45.1% (38.6%-51.8%) | 30.2% (24.4%-36.6%) |
| Intensity (how hard). n=214 | 8.9% (5.6%-13.2%) | 12.6% (8.7%-17.6%) | 29.4% (23.6%-35.8%) | 30.8% (24.9%-37.3%) | 18.2% (13.5%-23.8%) |
| Time or duration (how long). n=211 | 6.6% (3.9%-10.6%) | 6.2% (3.5%-10%) | 23.7% (18.3%-29.8%) | 37.9% (31.6%-44.6%) | 25.6% (20.1%-31.8%) |
| Type (aerobic/cardio, strength, endurance). n=213 | 5.2% (2.8%-8.8%) | 6.6% (3.8%-10.5%) | 19.7% (14.8%-25.4%) | 42.3% (35.8%-49%) | 26.3% (20.7%-32.5%) |
| **How often do the following items prevent you from promoting a physically active lifestyle in your patient management (apart from therapeutic / rehabilitative exercise)?** | | | | | |
| Lack of time. n=208 | 19.7% (14.7%-25.5%) | 28.8% (23%-35.3%) | 32.7% (26.6%-39.3%) | 14.4% (10.2%-19.7%) | 4.3% (2.2%-7.7%) |
| Lack of exercise guidance or counselling skills. n=206 | 30.6% (24.6%-37.1%) | 39.3% (32.8%-46.1%) | 23.8% (18.4%-29.9%) | 4.4% (2.2%-7.8%) | 1.9% (0.7%-4.6%) |
| Lack of remuneration for promoting physical activity. n=208 | 60.6% (53.8%-67%) | 25.5% (19.9%-31.7%) | 10.1% (6.6%-14.7%) | 3.4% (1.5%-6.5%) | 0.5% (0.1%-2.2%) |
| Lack of interest in promoting physical activity. n=207 | 72% (65.6%-77.8%) | 22.2% (17%-28.2%) | 4.3% (2.2%-7.8%) | 1.4% (0.4%-3.8%) | 0 |
| Belief that it would not change the patient’s behaviour. n=208 | 28.4% (22.6%-34.8%) | 25.5% (19.9%-31.7%) | 35.1% (28.9%-41.8%) | 6.3% (3.5%-10.2%) | 4.8% (2.5%-8.4%) |
| Belief that it would not be beneficial for the patient. n=208 | 55.8% (49%-62.4%) | 34.6% (28.4%-41.3%) | 9.6% (6.2%-14.2%) | 0 | 0 |
| Unaware of established community based physical activity programs (e.g., Tai Chi class, dance programs, walking groups, Get Healthy Program). n=205 | 29.3% (23.4%-35.8%) | 32.7% (26.5%-39.3%) | 27.3% (21.6%-33.7%) | 8.8% (5.5%-13.2%) | 2% (0.7%-4.6%) |
