# Supplementary material for: Insights into physical activity promotion among Australian chiropractors: a cross-sectional survey
Source: Chiropr Man Therap. 2024 Jun 14;32:22. doi: 10.1186/s12998-024-00543-2 (PMC11179190; doi:10.1186/s12998-024-00543-2)
Supplement: Supplementary file 2 — Supplementary Material 2 [file 12998_2024_543_MOESM2_ESM.docx]

**Supplementary Table 2. Perceptions of physical activity among Australian chiropractors.**

|  | **Strongly Agree** | **Agree** | **Not Sure** | **Disagree** | **Strongly Disagree** |
| --- | --- | --- | --- | --- | --- |
| **To what extent do you agree or disagree with the following statements:** | | | | | |
| Good health requires adding large muscle group strengthening activities (such as resistance or weight training) a few times per week. n=209 | 32.5% (26.5%-39.1%) | 49.3% (42.6%-56%) | 10.5% (6.9%-15.2%) | 6.7% (3.9%-10.7%) | 1% (0.2%-3%) |
| Any amount of physical activity counts. n=210 | 41.9% (35.4%-48.7%) | 46.7% (40%-53.4%) | 4.8% (2.5%-8.3%) | 6.7% (3.9%-10.6%) | 0 |
| Good health only requires 30 minutes of brisk walking on most days (total 150 to 300 minutes per week). n=210 | 7.6% (4.6%-11.8%) | 31.9% (25.9%-38.4%) | 11.4% (7.7%-16.3%) | 45.7% (39.1%-52.5%) | 3.3% (1.5%-6.4%) |
| Good health requires short bursts of exercise that gets your body warm and sweaty causing you to breathe heavily (total 75to 150 minutes per week). n=208 | 10.1% (6.6%-14.7%) | 52.9% (46.1%-59.6%) | 17.8% (13.1%-23.4%) | 17.8% (13.1%-23.4%) | 1.4% (0.4%-3.8%) |
| Good health requires being less sedentary by breaking up long periods of sitting as often as possible, substituted with movement of any intensity. n=209 | 54.1% (47.3%-60.7%) | 40.2% (33.7%-46.9%) | 2.9% (1.2%-5.8%) | 2.4% (0.9%-5.2%) | 0.5% (0.1%-2.2%) |
| Discussing the benefits of a physically active lifestyle with patients is an important part of the chiropractor's clinical role. n=210 | 71% (64.6%-76.8%) | 26.7% (21%-32.9%) | 1% (0.2%-3%) | 1% (0.2%-3%) | 0.5% (0.1%-2.2%) |
| Suggesting ways to increase a patient's daily physical activity is part of the chiropractor's clinical role. n=209 | 63.6% (57%-69.9%) | 32.5% (26.5%-39.1%) | 2.4% (0.9%-5.2%) | 1.4% (0.4%-3.8%) | 0 |
| As a chiropractor, I feel confident in giving general advice to patients about a physically active lifestyle. n=207 | 59.4% (52.6%-65.9%) | 37.2% (30.8%-43.9%) | 2.4% (0.9%-5.2%) | 1% (0.2%-3.1%) | 0 |
| As a chiropractor, I feel confident in suggesting specific physical activity programs to my patients. n=210 | 43.8% (37.2%-50.6%) | 41% (34.5%-47.7%) | 8.1% (5%-12.4%) | 7.1% (4.2%-11.2%) | 0 |
| As a chiropractor, I should be physically active to act as a role model for my patients. n=210 | 68.6% (62.1%-74.6%) | 29.5% (23.7%-35.9%) | 1% (0.2%-3%) | 1% (0.2%-3%) | 0 |
