# Supplementary material for: Insights into physical activity promotion among Australian chiropractors: a cross-sectional survey
Source: Chiropr Man Therap. 2024 Jun 14;32:22. doi: 10.1186/s12998-024-00543-2 (PMC11179190; doi:10.1186/s12998-024-00543-2)
Supplement: Supplementary file 3 — Supplementary Material 3 [file 12998_2024_543_MOESM3_ESM.docx]

**Supplementary Table 3. Feasibility of physical activity promotion among Australian chiropractors.**

|  | **Highly feasible** | **Somewhat feasible** | **Not sure** | **Not really feasible** | **Totally unfeasible** |
| --- | --- | --- | --- | --- | --- |
| **What kind of physical activity promotion is or would be feasible for you to deliver to your patients (beyond prescribing therapeutic /rehabilitative exercise)?** | | | | | |
| a. Brief exercise counselling integrated into your regular consultations. n=207 | 52.2% (45.4%-58.9%) | 34.8% (28.5%-41.4%) | 4.3% (2.2%-7.8%) | 8.7% (5.4%-13.1%) | 0% |
| b. Separate one-on-one consultations. n=207 | 21.7% (16.5%-27.7%) | 29.5% (23.6%-35.9%) | 16.4% (11.9%-21.9%) | 29.5% (23.6%-35.9%) | 2.9% (1.2%-5.9%) |
| c. Group sessions. n=208 | 15.4% (11%-20.8%) | 28.4% (22.6%-34.8%) | 15.4% (11%-20.8%) | 30.3% (24.3%-36.8%) | 10.6% (6.9%-15.3%) |
| d. Distribution of educational resources (e.g., Brochures). n=208 | 50% (43.2%-56.8%) | 36.5% (30.2%-43.2%) | 7.7% (4.6%-11.9%) | 4.8% (2.5%-8.4%) | 1% (0.2%-3%) |
| e. Recommending established community based physical activity programs (e.g., Tai Chi class, dance programs, walking groups, Get Healthy Program). n=205 | 69.8% (63.2%-75.7%) | 23.9% (18.5%-30.1%) | 4.4% (2.2%-7.9%) | 1.5% (0.4%-3.9%) | 0.5% (0.1%-2.3%) |
