# Supplementary material for: Insights into physical activity promotion among Australian chiropractors: a cross-sectional survey
Source: Chiropr Man Therap. 2024 Jun 14;32:22. doi: 10.1186/s12998-024-00543-2 (PMC11179190; doi:10.1186/s12998-024-00543-2)
Supplement: Supplementary file 4 — Supplementary Material 4 [file 12998_2024_543_MOESM4_ESM.docx]

**Supplementary Table 4. Familiarity with Physical Activity and Sedentary Behaviour guidelines among Australian chiropractors.**

|  |  | **n** | **% (CI)** |
| --- | --- | --- | --- |
| **Are you familiar with the current Australia's Physical Activity and Sedentary Behaviour Guidelines for Australian Adults -aged 18-64 years published by the Australian Government, Department of Health?** | | | |
|  | Not at all familiar | 75 | 36.6% (30.2%-43.3%) |
|  | Slightly familiar | 36 | 17.6% (12.8%-23.2%) |
|  | Somewhat familiar | 46 | 22.4% (17.1%-28.5%) |
|  | Moderately familiar | 34 | 16.6% (12%-22.1%) |
|  | Extremely familiar | 14 | 6.8% (4%-10.9%) |
