# Supplementary material for: Insights into physical activity promotion among Australian chiropractors: a cross-sectional survey
Source: Chiropr Man Therap. 2024 Jun 14;32:22. doi: 10.1186/s12998-024-00543-2 (PMC11179190; doi:10.1186/s12998-024-00543-2)
Supplement: Supplementary file 5 — Supplementary Material 5 [file 12998_2024_543_MOESM5_ESM.docx]

**Supplementary Table 5. Identifying all key components associated with Australia's current Physical Activity and Sedentary Behaviour Guidelines by Australian chiropractors.**

|  |  | **n** | **% (CI)** |
| --- | --- | --- | --- |
| **Which of the following best describes Australia's current Physical Activity and Sedentary Behaviour Guidelines for adults- aged 18-64 years:** | | | |
|  | The accumulation of at least 60 minutes of moderate to vigorous intensity physical activity daily. Additionally, muscle strengthening activities performed at least 3 days per week. Periods of prolonged sitting should be broken up as much as possible. | 8 | 3.9% (1.9%-7.3%) |
|  | The accumulation of 150 to 300 minutes (2.5 to5 hours) of moderate intensity physical activity or 75 to 150 minutes (1.25 to 2.5 hours) of vigorous intensity physical activity, or an equivalent combination of both each week. Additionally, muscle strengthening activities performed at least 2 days per week. Periods of prolonged sitting should be broken up as much as possible. | 94 | 46.3% (39.5%-53.2%) |
|  | The accumulation of at least 30 minutes of moderate to vigorous intensity physical activity on most, preferably all, days, which incorporates fitness, strength, balance and flexibility. Periods of prolonged sitting should be broken up as much as possible. | 59 | 29.1% (23.1%-35.6%) |
|  | Not sure | 42 | 20.7% (15.6%-26.7%) |
