# Supplementary material for: Insights into physical activity promotion among Australian chiropractors: a cross-sectional survey
Source: Chiropr Man Therap. 2024 Jun 14;32:22. doi: 10.1186/s12998-024-00543-2 (PMC11179190; doi:10.1186/s12998-024-00543-2)
Supplement: Supplementary file 6 — Supplementary Material 6 [file 12998_2024_543_MOESM6_ESM.docx]

**Supplementary Table 6. Australia chiropractors own physical activity engagement and meeting the Physical Activity and Sedentary Behaviour guidelines.**

|  |  | **n** | **% (CI)** |
| --- | --- | --- | --- |
| **About your own physical activity: How would you compare your current level of physical activity to other Australians of your sex and similar age?** | | | |
|  | Much more active | 77 | 37.7% (31.3%-44.5%) |
|  | Slightly more active | 74 | 36.3% (29.9%-43%) |
|  | About the same | 30 | 14.7% (10.4%-20%) |
|  | Slightly less active | 16 | 7.8% (4.7%-12.1%) |
|  | Much less active | 5 | 2.5% (0.9%-5.3%) |
|  | Prefer not to answer | 2 | 1% (0.2%-3.1%) |
| **In the last 6-months did you typically 'Accumulate 150 to 300minutes (2 ½ to 5 hours) of moderate intensity physical activity or 75 to 150 minutes (1 ¼ to 2 ½ hours) of vigorous intensity physical activity, or an equivalent combination of both moderate and vigorous activities, each week.'** | | | |
|  | Yes | 169 | 83.3% (77.7%-87.9%) |
|  | No | 32 | 15.8% (11.3%-21.2%) |
|  | Prefer not to answer | 2 | 1% (0.2%-3.1%) |
| **In the last 6-months did you typically 'perform muscle strengthening activities on at least 2 days each week?'** | | | |
|  | Yes | 155 | 77.1% (70.9%-82.5%) |
|  | No | 46 | 22.9% (17.5%-29.1%) |
|  | Prefer not to answer | 0 | 0% |
