# Supplementary material for: Insights into physical activity promotion among Australian chiropractors: a cross-sectional survey
Source: Chiropr Man Therap. 2024 Jun 14;32:22. doi: 10.1186/s12998-024-00543-2 (PMC11179190; doi:10.1186/s12998-024-00543-2)
Supplement: Supplementary file 7 — Supplementary Material 7 [file 12998_2024_543_MOESM7_ESM.docx]

**Study survey questionnaire – adapted to a chiropractic setting:**

**Physical Activity in Chiropractic Practice Questionnaire**

Physical activity includes any activity from a low intensity level, such as walking, cycling, or swimming, to a high intensity level, such as running, cycling and playing a competitive sport. Physical activity is different to prescribing therapeutic or rehabilitative exercise.

1. **General questions about your practice**:

| a. Average number of patient visits per week: | | ____________ | |
| --- | --- | --- | --- |
| b. Number of years in practice: | | ____________ | |
| c. Your gender: | |  Male  Female  Intersex  Rather not say | |
| d. Your age in years: | |  <34  35-44  45-54  >54  55-64  >64 | |
| e. Average number of hours worked per week: | | ____________ | |
| 1. The postal code of the main clinic where you work: 2. Other qualifications / postgrad qualifications: | | ____________  ____________ | |
| f. In what area of chiropractic do you practice? (*please select all that apply to you)* |  Older people (65 years and over)   Children (4-18 years)   Children (up to 3 years) |  Pregnant women   Working age adults   Athletes or sports people |  Work/traffic related injuries   Post-surgical rehabilitation   Other (please specify) |
| g. In what kind of clinical environment do you work? | |  Private practice   Academia (research and education)   Both private practice and academia   Other | |

1. **What percentage of your patients did you recommend** **having a more physically active lifestyle (apart from therapeutic / rehabilitative exercise) in the last month?** (*please tick one*)

|  Never   Rarely, in less than 10% of patients in the last month   Occasionally, in about 30% of patients in the last month   Sometimes, in about 50% of patients in the last month |  Frequently, in about 70% of patients in the last month   Usually, in about 90% of patients in the last month   Every time |
| --- | --- |

1. **How often do you perform pre-exercise screening (e.g. baseline bodyweight, heart rate and blood pressure etc.) on your patients prior to recommending physical activity?** (*please select one*)

| **Never** | **Rarely** | **Sometimes** | **Often** | **Very often** |
| --- | --- | --- | --- | --- |
| 1 | 2 | 3 | 4 | 5 |

1. **How frequently do you recommend or prescribe the following;** (*Select one for each statement)*

|  | **Never** | **Rarely** | **Sometimes** | **Often** | **Very often** |
| --- | --- | --- | --- | --- | --- |
| 1. Aerobic exercise (i.e. endurance training) | 1 | 2 | 3 | 4 | 5 |
| 1. Resistance exercise (i.e. strength training) | 1 | 2 | 3 | 4 | 5 |
| 1. Flexibility training (i.e. stretching) | 1 | 2 | 3 | 4 | 5 |
| 1. Balance training | 1 | 2 | 3 | 4 | 5 |

1. **How frequently do you determine or quantify your prescription of physical activity under the FITT principles (Frequency, Intensity, Time and Type);** (*Select only one for each statement)*

|  | **Never** | **Rarely** | **Sometimes** | **Often** | **Very often** |
| --- | --- | --- | --- | --- | --- |
| a. Frequency (how often) | 1 | 2 | 3 | 4 | 5 |
| b. Intensity (how hard) | 1 | 2 | 3 | 4 | 5 |
| c. Time or duration (how long) | 1 | 2 | 3 | 4 | 5 |
| d. Type (aerobic/cardio, strength, endurance) | 1 | 2 | 3 | 4 | 5 |

1. **To what extent do you agree or disagree with the following statements:** (*select only one for each statement)*

|  | **Strongly agree** | **Agree** | **Not Sure** | **Disagree** | **Strongly disagree** |
| --- | --- | --- | --- | --- | --- |
| a. Good health requires adding large muscle group strengthening activities (such as resistance or weight training) a few times per week. | 1 | 2 | 3 | 4 | 5 |
| b. Any amount of physical activity counts. | 1 | 2 | 3 | 4 | 5 |
| c. Good health only requires 30 minutes of brisk walking on most days (total 150 to 300 minutes per week). | 1 | 2 | 3 | 4 | 5 |
| d. Good health requires short bursts of exercise that gets your body warm and sweaty causing you to breathe heavily (total 75 to 150 minutes per week). | 1 | 2 | 3 | 4 | 5 |
| e. Good health requires being less sedentary, breaking up long periods of sitting as often as possible, substituted with movement of any intensity. | 1 | 2 | 3 | 4 | 5 |
| f. Discussing the benefits of a physically active lifestyle with patients is an important part of the chiropractor’s clinical role | 1 | 2 | 3 | 4 | 5 |
| g. Suggesting ways to increase patient’s daily physical activity is part of the chiropractor’s clinical role | 1 | 2 | 3 | 4 | 5 |
| h. As a chiropractor, I feel confident in giving general advice to patients about a physically active lifestyle | 1 | 2 | 3 | 4 | 5 |
| i. As a chiropractor, I feel confident in suggesting specific physical activity programs to my patients | 1 | 2 | 3 | 4 | 5 |
| j. As a chiropractor, I should be physically active to act as a role model for my patients | 1 | 2 | 3 | 4 | 5 |

1. **How often do the following items prevent you from promoting a physically active lifestyle in your patient management (apart from prescribing therapeutic / rehabilitative exercise)?** *(select only one for each statement)*

|  | **Never** | **Rarely** | **Sometimes** | **Often** | **Very often** |
| --- | --- | --- | --- | --- | --- |
| 1. Lack of time | 1 | 2 | 3 | 4 | 5 |
| 1. Lack of ‘exercise guidance or counselling’ skills | 1 | 2 | 3 | 4 | 5 |
| 1. Lack of remuneration for promoting physical activity | 1 | 2 | 3 | 4 | 5 |
| 1. Lack of interest in promoting physical activity | 1 | 2 | 3 | 4 | 5 |
| 1. Belief that it would not change the patient’s behaviour | 1 | 2 | 3 | 4 | 5 |
| 1. Belief that it would not be beneficial for the patient | 1 | 2 | 3 | 4 | 5 |
| 1. Unaware of established community based physical activity programs (e.g. Tai Chi class, dance programs, walking groups, Get Healthy Program) | 1 | 2 | 3 | 4 | 5 |
| 1. Other: ___________ | 1 | 2 | 3 | 4 | 5 |

1. **What kind of physical activity promotion is or would be feasible for you to deliver to your patients (beyond prescribing therapeutic / rehabilitative exercise)?** *(select only one for each statement)*

|  | **Highly feasible** | **Somewhat feasible** | **Not sure** | **Not really feasible** | **Totally unfeasible** |
| --- | --- | --- | --- | --- | --- |
| 1. Brief exercise counselling integrated into your regular consultations | 1 | 2 | 3 | 4 | 5 |
| 1. Separate one-on-one consultations | 1 | 2 | 3 | 4 | 5 |
| 1. Group sessions | 1 | 2 | 3 | 4 | 5 |
| 1. Distribution of educational resources (e.g. Brochures) | 1 | 2 | 3 | 4 | 5 |
| 1. Recommending established community based physical activity programs (e.g. Tai Chi class, dance programs, walking groups, Get Healthy Program) |  |  |  |  |  |
| 1. Other: ____________ | 1 | 2 | 3 | 4 | 5 |

|  | **Not at all familiar** | **Slightly familiar** | **Somewhat familiar** | **Moderately familiar** | **Extremely familiar** |
| --- | --- | --- | --- | --- | --- |
| **Are you familiar with the current Australia’s Physical Activity and Sedentary Behaviour Guidelines for Australian Adults – aged 18-64 years published by the Australian Government, Department of Health?** | 1 | 2 | 3 | 4 | 5 |

**9a. Which of the following best describes Australia’s current Physical Activity and Sedentary Behaviour Guidelines for adults – aged 18-64 years:**

1. The accumulation of at least 60 minutes of moderate to vigorous intensity physical activity daily. Additionally, muscle strengthening activities performed at least 3 days per week. Periods of prolonged sitting should be broken up as much as possible.
2. The accumulation of 150 to 300 minutes (2.5 to 5 hours) of moderate intensity physical activity or 75 to 150 minutes (1.25 to 2.5 hours) of vigorous intensity physical activity, or an equivalent combination of both each week. Additionally, muscle strengthening activities performed at least 2 days per week. Periods of prolonged sitting should be broken up as much as possible.
3. The accumulation of at least 30 minutes of moderate to vigorous intensity physical activity on most, preferably all, days, which incorporates fitness, strength, balance and flexibility. Periods of prolonged sitting should be broken up as much as possible.
4. Not sure

1. **About your own physical activity: How would you compare your current level of physical activity to other Australians of your sex and similar age?** (*please tick one) Participants must answer Q10 to see Q11 & Q12.*

|  Much more active |  Slightly more active |  About the same |  Slightly less active |  Much less active |  Prefer not to answer |
| --- | --- | --- | --- | --- | --- |

1. **In the last 6-months did you typically ‘Accumulate 150 to 300 minutes (2 ½ to 5 hours) of moderate intensity physical activity or 75 to 150 minutes (1 ¼ to 2 ½ hours) of vigorous intensity physical activity, or an equivalent combination of both moderate and vigorous activities, each week?’**

 Yes No  Prefer not to answer

1. **In the last 6-months did you typically ‘perform muscle strengthening activities on at least 2 days each week?’**

 Yes No  Prefer not to answer
