# Supplementary material for: Insights into physical activity promotion among Australian chiropractors: a cross-sectional survey
Source: Chiropr Man Therap. 2024 Jun 14;32:22. doi: 10.1186/s12998-024-00543-2 (PMC11179190; doi:10.1186/s12998-024-00543-2)
Supplement: Supplementary file 8 — Supplementary Material 8 [file 12998_2024_543_MOESM8_ESM.docx]

**Supplementary Table 7. Exploratory logistic regression models identifying Australian chiropractor characteristics associated with encouraging patients to be more physically active, referenced against those who infrequently recommend physical activity.**

|  | | | **OR** | **95% CI** | **P-Value** |
| --- | --- | --- | --- | --- | --- |
| **Practitioner and Patients** | | |  |  |  |
| Age ≤45 years | | | 1.15 | 0.65-2.04 | 0.637 |
| Usual weekly hours ≤30 | | | 1.06 | 0.6-1.87 | 0.834 |
| Average weekly patient ≤60 | | | 1.18 | 0.68-2.06 | 0.56 |
| Years in practice ≤10 | | | 0.88 | 0.51-1.54 | 0.663 |
| Older people (65 years and over) | | | 0.5 | 0.22-1.11 | 0.087 |
| Working age adults | | | 0.29 | 0.03-2.44 | 0.253 |
| Athletes or sports people | | | 1.69 | 0.84-3.4 | 0.145 |
| Work/traffic related injuries | | | 0.93 | 0.53-1.62 | 0.789 |
| Post-surgical rehabilitation | | | 1.12 | 0.63-2.02 | 0.696 |
| Other | | | 1.07 | 0.45-2.53 | 0.88 |
| **Practice** | | |  |  |  |
| Other | | | 1.42 | 0.27-7.49 | 0.681 |
| **How frequently do you recommend or prescribe the following** | | |  |  |  |
| Flexibility training (i.e., stretching). n=212 | | | 1.19 | 0.65-2.18 | 0.571 |
| Balance training. n=214 | | | 1.53 | 0.87-2.68 | 0.142 |
| **How often do the following items prevent you from promoting a physically active lifestyle in your patient management (apart from therapeutic / rehabilitative exercise)?** | | | | | |
| Lack of time. n=208 | | | 0.49 | 0.24-1 | 0.051 |
| Lack of exercise guidance or counselling skills. n=206 | | | 0.44 | 0.14-1.35 | 0.149 |
| Lack of remuneration for promoting physical activity. n=208 | | | 3.94 | 0.48-32.64 | 0.204 |
| Lack of interest in promoting physical activity. n=207 | | | 1.09 | 0.1-12.24 | 0.944 |
| Belief that it would not change the patient’s behaviour. n=208 | | | 0.55 | 0.23-1.32 | 0.18 |
| Unaware of established community based physical activity programs (e.g., Tai Chi class, dance programs, walking groups, Get Healthy Program). n=205 | | | 0.74 | 0.3-1.83 | 0.514 |
| **To what extent do you agree or disagree with the following statements:** |  |  | | | |
| Any amount of physical activity counts. n=210 | | | 0.91 | 0.37-2.24 | 0.836 |
| Good health only requires 30 minutes of brisk walking on most days (total 150 to 300 minutes per week). n=210 | | | 1.12 | 0.62-2 | 0.712 |
| Good health requires short bursts of exercise that gets your body warm and sweaty causing you to breathe heavily (total 75 to 150 minutes per week). n=208 | | | 0.83 | 0.46-1.51 | 0.543 |
| Good health requires being less sedentary by breaking up long periods of sitting as often as possible, substituted with movement of any intensity. n=209 | | | 1.9 | 0.59-6.11 | 0.283 |
| As a chiropractor, I should be physically active to act as a role model for my patients. n=210 | | | 5.7 | 0.58-55.84 | 0.135 |
| **What kind of physical activity promotion is or would be feasible for you to deliver to your patients (beyond prescribing therapeutic /rehabilitative exercise)?** | | | | | |
| Recommending established community based physical activity programs (e.g., Tai Chi class, dance programs, walking groups, Get Healthy Program). n=205 | | | 1.64 | 0.53-5.07 | 0.393 |
| Are you familiar with the current Australia's Physical Activity and Sedentary Behaviour Guidelines for Australian Adults - aged 18-64 years published by the Australian Government, Department of Health? n=205 | | | 2.9 | 1.32-6.41 | 0.008 |

Statistically non-significant findings
